# Spatial Release from Masking for Tones and Noises in a Soundfield under Conditions Where Targets and Maskers Are Stationary or Moving

**DOI:** 10.3390/audiolres12020013

**Published:** 2022-02-23

**Authors:** M. Torben Pastore, William A. Yost

**Affiliations:** College of Health Solutions, Arizona State University, Phoenix, AZ 85004, USA; william.yost@asu.edu

**Keywords:** spatial hearing, spatial release from masking

## Abstract

Stationary visual targets often become far more salient when they move against an otherwise static background–the so-called “pop out” effect. In two experiments conducted over loudspeakers, we tested for a similar pop-out effect in the auditory domain. Tone-in-noise and noise-in-noise detection thresholds were measured using a 2-up, 1-down adaptive procedure under conditions where target and masker(s) were presented from the same or different locations and when the target was stationary or moved via amplitude-panning. In the first experiment, target tones of 0.5 kHz and 4 kHz were tested, maskers (2–4, depending on the condition) were independent Gaussian noises, and all stimuli were 500-ms duration. In the second experiment, a single pink noise masker (0.3–12 kHz) was presented with a single target at one of four bandwidths (0.3–0.6 kHz, 3–6 kHz, 6–12 kHz, 0.3–12 kHz) under conditions where target and masker were presented from the same or different locations and where the target moved or not. The results of both experiments failed to show a decrease in detection thresholds resulting from movement of the target.

## 1. Introduction

In everyday environments, listeners and sound sources often move. While much is known about auditory detection under stationary conditions over headphones, far less is known about sound-source detection over loudspeakers in a soundfield and the effects, if any, of sound-source motion on sound-source detection. It has been shown that listeners often enjoy an improved ability to detect or understand speech when the target stimulus is presented from a different location than other simultaneous interfering signals (maskers). This improvement in performance over conditions where target and masker are presented from the same location is called spatial release from masking (SRM) and is often measured as the difference, in decibels, between the masked thresholds in the two conditions.

### 1.1. Speech Identification

Pastore and Yost [1] tested for an effect of auditory motion on speech identification in a soundfield. They asked listeners to identify a word spoken by a female voice presented simultaneously with other words spoken by 2 or 4 male voices under conditions where target and maskers were presented from the same location or where they were all at different locations, with the maskers symmetrically placed around the listener. In half of the presentations, the target word was dynamically panned between $\pm 20^{\circ }$ in front of the listener, so that the target word perceptually moved and in the other half, the target was stationary. Maskers were stationary for all presentations. Spatial release from masking was then compared between conditions. Pastore and Yost [1] found no change in performance that was systematically related to target movement, and therefore concluded that, at least for word identification, there is unlikely to be an auditory correlate to the visual ‘pop out’ effect.

Muñoz et al. [2] investigated spatial release from masking using simulated room acoustic conditions reproduced over headphones, using non-individualized head-related transfer functions (HRTFs). Both young and elderly subjects were tested. Maskers were either (1) stationary, (2) moved around the listener along a circular trajectory, or (3) moved away from the listener along a radial trajectory (i.e., out from the center away from the listener). Testing was done under simulated anechoic conditions and simulated reverberant room acoustic conditions. Under simulated anechoic conditions, when maskers were moved around the listener along a circular trajectory, SRM was less than when the masker was stationary at a variety of locations. That is, movement of the maskers had a small but reliable negative effect on SRM. By contrast, when a masker was moved away from the listener along a radial trajectory, SRM was greater than when the masker was stationary by approximately 1.5 dB. Perhaps unsurprisingly, SRM decreased in general as reverberation was introduced for all conditions. In general, all these effects were more pronounced in the elderly subjects than in the younger subjects. Since Muñoz et al. used non-individualized head-related transfer functions and there was no compensation for head movements, stimuli may or may not have been perceptually fully externalized. See [3,4,5,6] for more on the role of HRTFs, reverberation, and head movements in perceived externalization and distance perception.

It may be that there is some effect of sound-source motion on listeners’ detection of the signal’s presence that is lost in the more complex, and therefore ‘noisier’ task of speech identification. In speech identification, concurrent talkers or other sounds can diminish a listener’s ability to identify speech in two overall ways. First, the spectro-temporal overlap of the target speech and maskers may reduce the audibility of the target speech. Second, the similarity of the target speech and its maskers, the overall increased complexity of the soundfield, or any other reduction in intelligibility that occurs that is not the result of energetic masking may have affected speech identification in ways that are unclear. This ‘informational masking’ is usually thought of as affecting ‘higher processing’ such as attention, auditory stream formation, speech processing, etc. In the case of Pastore and Yost [1], informational masking at the level of speech processing may have obscured any additional spatial release from masking, in terms of detection, that might occur as a result of target motion.

### 1.2. Sound Detection

While there has been considerable study of listeners’ ability to detect the movement of sound sources in a soundfield (e.g., [7,8,9], and see [10,11,12] for reviews), few studies have tested for an improvement in listeners’ ability to detect the presence of a target sound under conditions where the sound source moved. A few such experiments have been conducted over headphones. For example, Wilcott and Gales [13] measured detection thresholds for a low-pass filtered noise dichotically-presented with interaural phase differences that were static, or changed in a linear step-wise manner, to simulate a moving sound source. The target noise was masked by two independent noises, one presented to each ear. The masked threshold was essentially the same regardless of whether the interaural phase of the target remained static or changed during presentation, leading the authors to conclude that auditory motion was unlikely to improve detection. Grantham and Luethke [14] conducted a similar experiment, also over headphones, presenting 400- or 800-Hz tones together with a noise masker that was either diotic, interaurally uncorrelated, or interaurally phase reversed. For conditions that simulated auditory motion, the left and right ear inputs were set to slightly different frequencies so the interaural phase difference (IPD) changed over the duration of the stimulus, again simulating motion. As with Wilcott and Gales [13], dynamic IPDs did not lead to a substantive change in detection thresholds.

These early reports do not suggest that motion is a strong cue for auditory detection for low-frequency stimuli. However, neither paper reported data for high frequency stimuli where interaural level differences (ILDs), instead of ITDs, are the dominant spatial auditory cue, nor did either experiment present broadband stimuli. Also, both experiments were conducted over headphones, and there are surprisingly few sound source detection studies conducted over loudspeakers, where ITDs and ILDs are present in ‘natural’ combinations that lead to externalization of auditory images as opposed to the ‘in the head’ sensation elicited by most headphone stimuli, where ITDs or ILDs are often manipulated separately as opposed to in the ratios they present with in a soundfield (e.g., see [15]). While some simulation studies (e.g., Muñoz [2]) have presented stimuli that are spatialized using non-individualized head-related transfer functions (HRTFs) so that the ratios of ITD vs. ILD are more natural, the monaural spectral details of the presented sounds do not accurately reflect the individual’s HRTFs. In these cases, listeners typically report sounds that are weakly externalized if at all, and localization is negatively impacted, primarily in terms of elevation and front-back localization (e.g., see [16,17]). Perception of auditory motion may occur at levels well higher than the auditory periphery. If this were the case, it is possible that auditory motion would be more salient for externalized, palpable auditory objects than for headphone stimuli.

Xiao and Grantham [18] tested for a difference in SRM for moving target stimuli in a soundfield over loudspeakers by presenting moving targets made of high- and low-frequency tones, and a broadband noise, in a background of two decorrelated noises placed at the extreme ends of the targets’ fairly small range of motion, at approximately $\pm 12^{\circ }$. The two maskers were presented at a very low intensity of 40 dB and were played for the entire duration of the experiment. Mention is also made of an HVAC system that made audible sound where the experiment was performed. While Xiao and Grantham [18] found that target motion had no effect on detection, it may be that the very low level at which stimuli were presented, especially in the presence of HVAC noise, could have obscured some effect of target motion. Not having a rest from the maskers between presentations may also have reduced listeners’ attention to the task. It may also be that a greater range of motion than that necessary for the detection of sound-source motion per se was necessary for the motion to affect detection of the target in a masking situation. Our study sought to replicate and affirm the results of [18] under conditions where there could be no doubt as to the interpretation of the results. Finally, we sought to learn if movement of maskers would lead to a different outcome than movement of the target, as found by Muñoz et al. [2]

We conducted two experiments investigating the effect of target motion on auditory detection. In order to produce data that are at least somewhat comparable to those of Pastore and Yost [1], pink noise maskers, with a power spectrum that more closely approximates that of speech, were used. All experiments presented in this paper were conducted over loudspeakers in a sound-deadened room, described in further detail below in the Methods section. The listeners’ task was to detect the presence of a target stimulus (tones at 500 and 4000 Hz in Experiment 1 and various bandwidths of noise in Experiment 2) when presented with two or more noise maskers. The location and movement of the target and maskers were manipulated to probe the effect, if any, of sound source movement on listeners’ performance in an auditory detection task.

## 2. Materials and Methods

### 2.1. Subjects

Eleven listeners between the ages of 18 and 31 (mean ≈ 21 yrs.) were tested in Experiment 1 and 10 listeners (6 female in both experiments) were tested in Experiment 2, with 9 participating in both experiments. All listeners reported normal hearing; this was verified with audiometric testing (all listeners <10-dB hearing level at octave frequencies from 125–8000 Hz.) No listener had previously participated in any psychoacoustic experiment using the same testing paradigm. The procedures used in these experiments were approved by the Arizona State University Institutional Board for the Protection of Human Subjects (IRB).

### 2.2. Listening Room

The listening room was the same used for [1,19]. The room measures $15^{\prime }\times 12^{\prime }\times 10^{\prime }$ (4.57 m × 3.66 m × 3.05 m) and is lined with $4^{\prime \prime }$ (10.2 cm) thick acoustic foam on all six surfaces, resulting in a wideband reverberation time (${\mathrm{RT}}_{60}$) of 102 ms. Twenty-four loudspeakers (Boston Acoustics 100X, Peabody, MA, USA) are located on a $10^{\prime }$ diameter azimuth circle at pinna height. Listeners were seated in the middle of the circular array and were monitored via intercom and camera from an adjoining control room. Listeners were asked to keep their heads stationary and face the center loudspeaker, which had a red circle on it. Listeners were closely monitored to be sure they followed directions.

### 2.3. Procedure

All testing was blocked by stimulus condition and target tone frequency. To minimize listener confusion, stimuli were presented in blocks so that all panning was in one direction for one block and the other for the next block for both panned and static stimuli. Blocks were in random order and, in the case of dynamic panning, listeners were alerted via a computer monitor which side the target would be on or coming from. Listeners used a keypad to indicate whether they heard the tonal target or not for each presentation.

The 2-up, 1-down adaptive procedure included 12 total reversals, and worked as follows: for the first first 2 reversals, the level of the target was decreased by 2 dB after 2 consecutive correct responses. For each incorrect response, the level of the target was increased by 2 dB. For the next 10 reversals, the level of the target was decreased by 1 dB after 2 consecutive correct responses. For each incorrect response, the level of the target was increased by 1 dB. After the 12th reversal, the 9 target stimulus values for the previous 9 reversals were averaged to arrive at the listener’s threshold for that condition. The results of two such tracks were averaged together for the listener’s final average threshold.

Pilot experiments with all listeners confirmed that they could reliably discriminate panned sound source motion (and its direction) for both the 500-Hz and 4000-Hz stimuli when presented on their own without interfering noise. Pastore and Yost [1] included a control experiment where they presented speech stimuli panned to simulate the same rate of perceived sound source motion as in the main experiments in this paper. These stimuli were presented in the presence of interfering talkers, and listeners’ detection of the direction of sound source motion ranged from 69% to 100% (mean of 84% across six listeners).

### 2.4. Data Analysis

Comparisons between the five conditions tested in Experiment 1 were within-subjects analyses of variance (ANOVA) with a significance criterion of α=0.05. The results of Experiment 2 were analyzed using pairwise (i.e., within subjects) *t*-tests between the baseline co-located target and masker condition (CO) and the two experimental conditions described below in Experiment 2. Because both *t*-tests included the baseline condition (CO), the significance criterion was adjusted for family-wise error using the Bonferroni correction (α=0.05/2). In all cases the null hypothesis was that target or masker movement would result in no statisically significant change in detection thresholds across listeners.

## 3. Experiment 1

### 3.1. Stimuli

Experiment 1 presented tone-in-noise stimuli that were gated on and off together. While the greatest masking level differences over headphones have been found for tones that are presented against continuous noise–i.e., a masker fringe that both precedes and follows the tone to be detected, Yost [20] showed that roughly the same thresholds and binaural masking level differences (BMLD) were measured when target and noise were gated together with a total duration of 500 ms, so this duration was used in Experiment 1.

**Figure 1:**
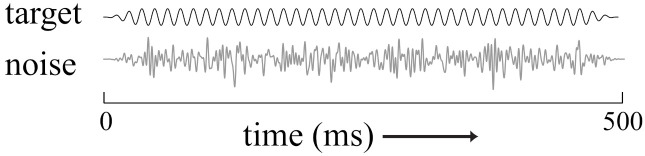
A schematic represtentation of the stimuli presented in Experiment 1. Details are provided in the text.

Figure 1 shows a schematic representation of the stimuli for Experiment 1. Stimuli consisted of pink noise maskers presented at 60 dB SPL, and a target sine tone of either 0.5 kHz or 4 kHz that was presented at 70 dB SPL at the beginning of each adaptive track. Both target and maskers were 500-ms duration with 20-ms $\cos^{2}$ onset and offset ramps. Fresh noise tokens were used for each presentation. For panned stimuli, the stimulus was presented simultaneously from loudspeakers at $\pm 30^{\circ }$. The relative voltages at the two loudspeakers were adjusted according to the sine law of amplitude panning (e.g., [21]). This resulted in a perceived ‘phantom’ sound source with constant overall power that either moved from $+20^{\circ }$ to $+20^{\circ }$, or vice-versa (dynamic panning) or was presented from a single stationary location at either $+20^{\circ }$ to $+20^{\circ }$ (static panning). Grantham [22] demonstrated that, for low-frequency tones, the sine law of panning produces phantom sound sources with the same interaural differences as actual sources and listeners are unable to distinguish between phantom and actual sound sources. For an explanation of how, in stereo panning, level differences between the two loudspeakers translate to interaural time differences at the listener’s ears, see Blauert [23], p. 214.

### 3.2. Conditions

**Figure 2:**
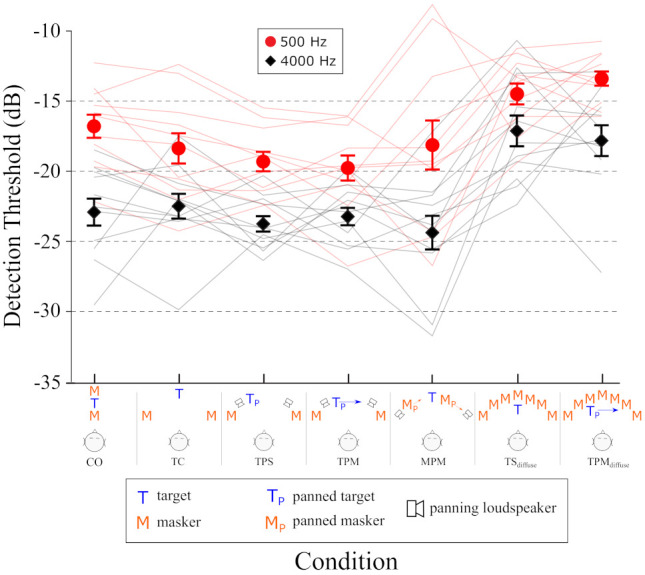
Mean tone–in–noise detection thresholds across 11 listeners, in terms of the difference between the levels of the maskers and the target, in decibels. Negative thresholds mean that the target energy was less than that of the maskers. Error bars indicate the variability across listeners as ±1 standard error of the mean. Individual data are represented with thin lines connecting performance for each condition, and are color coded the same as the mean data. Stimulus conditions are schematically illustrated along the horizontal axis.

The first 5 conditions, described below and schematically in Figure 2, were analogous to those tested in [1].

- **CO, Co-located condition:** The masking pink noise was co-located with the tone at the loudspeaker located directly in front of the listener at 0${}^{\circ }$.
- **TC, Target Centered**: Two independent pink noise maskers were presented, one from the loudspeaker at +45${}^{\circ }$ and the other from the loudspeaker at $-45^{\circ }$. The tone was presented from the loudspeaker at 0${}^{\circ }$. The CO and TC conditions together served to calculate the standard baseline value of spatial release from masking for comparisons with target panning conditions.
- **TPM, Target Panned Moving**: Independent pink noise maskers were presented as in the TC condition, from loudspeakers at +45${}^{\circ }$ and $-45^{\circ }$. The tone was amplitude panned, from the beginning of the stimulus to the end, so that a phantom sound source moved from +20${}^{\circ }$ to $-20^{\circ }$ or vice versa. Loudspeakers at $\pm 30^{\circ }$ were used to present the panned noises. If motion of a sound source improved detection, then this condition should elicit a greater release from masking than did the TC condition above.
- **TPS, Target Panned Static**: Independent pink noise maskers were presented as in the TC condition, from loudspeakers at $\pm 45^{\circ }$. The tone was panned between loudspeakers at $\pm 30^{\circ }$ so that its phantom image would be perceived at either $+20^{\circ }$ or $-20^{\circ }$. This condition acted as a control to determine what, if any, effect the off-center, panned presentation of the target had in the TPM condition as compared with the TC condition.
- **MPM, Masker Panned Moving**: The target was presented from the 0${}^{\circ }$ front loudspeaker and the two pink noise maskers were panned, in the same direction, from left to right between speakers at $\pm 60^{\circ }$, or vice-versa. The perceived masker locations were expected to migrate from $60^{\circ }\to 25^{\circ }$ and $-25^{\circ }\to -60^{\circ }$. This condition sought to determine if masker motion might make detection of a stationary target more or less difficult.

Two additional conditions were added. For the TPM dynamically-panned target condition, the target is at times closer to one masker than the other (when it is off to one side or the other), and at times spatially separated from both maskers (when it is near the center). It is possible that this could have had some effect, so a secondary pair of control conditions were employed so that the target was always essentially co-located with at least one loudspeaker at all times (these are the TS${}_{D}$ and TPM${}_{D}$ conditions, described below). Given that more loudspeakers were presenting independent noises, each at the same intensity as in the first 5 conditions, we expected the threshold measured in these two ‘diffuse’ conditions to be higher, because there is approximately 5.44 dB more masker energy as compared with the other conditions with 2 maskers (assuming incoherent addition of independent noises of equal rms amplitude). The comparison between the TS${}_{D}$ and TPM${}_{D}$ conditions should show if there is any improvement in detection due to target motion *per se*.

- **TS**_*D*_, **Diffuse Static**: The target was statically presented from any one of the loudspeakers at $-30^{\circ }$, $-15^{\circ }$, $0^{\circ }$, $+15^{\circ }$, $+30^{\circ }$ at random. All 7 loudspeakers from $-45^{\circ }$ to $+45^{\circ }$ simultaneously presented pink noise maskers, with each loudspeaker presenting an independent noise. This condition is analogous to the TC condition above.
- **TPM**_*D*_, **Diffuse Moving**: The same as ${\mathrm{TS}}_{D}$ above, but the target tone was panned between loudspeakers at $\pm 30^{\circ }$ from left to right or right to left at random, in the same way as it was in the TPM condition above. This condition is analogous to the TPM condition above.

### 3.3. Results

Figure 2 shows the mean tone-in-noise detection thresholds averaged across 11 listeners. Thresholds were systematically lower for 4-kHz tonal targets than for 500-Hz targets. This is unsurprising, given (1) pink noise maskers were used, so the power of the masker at 4 kHz was approximately 12.5% that of the masker power at 500 Hz (assuming 1/*f* power distribution) and (2) equal loudness contours show that listeners are more sensitive to stimulus energy around 4 kHz than 500 Hz. A within-subjects ANOVA on thresholds, measured across-subjects, indicated that there was no statistically significant difference (α=0.05) between the five main conditions. Between-subjects variability, the difference in performance between different listeners, was quite high, and no clear trend across listeners, as a function of stimulus conditions, could be identified (see the individual data indicated with thin lines in red (500 Hz target) and black (4000 Hz target)). That is, some demonstrated a release from masking for conditions where others experienced the opposite. Comparing thresholds for the TC and MPM conditions shows little difference for both high and low frequencies. It appears to make little difference, in terms of detection, whether the target or the maskers move.

**Figure 3:**
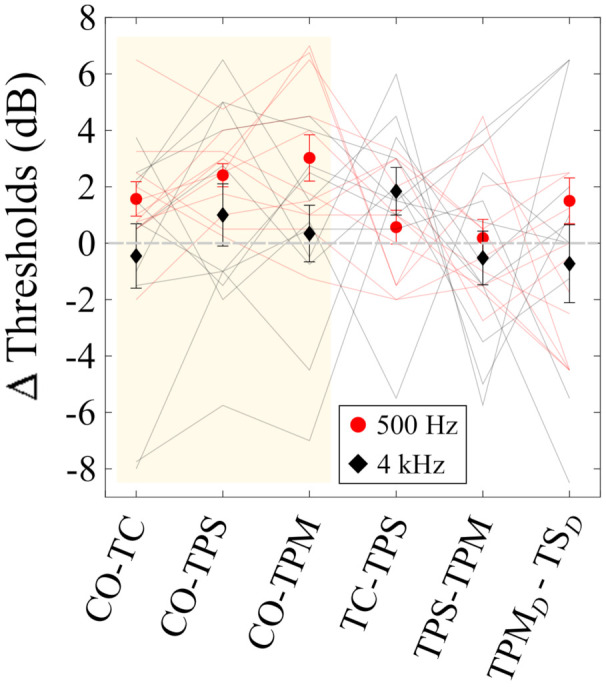
Mean change in tone–in–noise detection thresholds across 11 listeners. Error bars indicate ±1 standard error of the mean. The first 3 comparisons, indicated by the shaded area, represent measures of Spatial Release from Masking. The remaining data show comparisons of thresholds between other conditions. Individual data are represented with thin lines connecting performance for each condition, and are color coded the same as the mean data.

Figure 3 shows calculated spatial release from masking (SRM), along with several other differences in thresholds between conditions. The mean and standard error of the mean across listeners are shown with red filled circles (500 Hz) and black diamonds (4000 Hz). Individual listeners’ data are shown with thin lines, color coded for target frequency the same as the the solid symbols. Note that the systematic difference in thresholds between high and low frequency tonal targets is not expected for SRM calculations, since comparisons are now between different spatial configurations at the same stimulus frequencies. However, low-frequency stimuli are localized primarily on the basis of ITDs, whereas ILDs are the dominant auditory spatial cue at high frequencies (e.g., [24]).

The mean SRM, shown in the first three comparisons and shaded in gray, was ≈3 dB or less for the 500-Hz target tone and ≈1.75 dB or less for the 4 kHz target tone. Between-subjects variability was quite high relative to the magnitude of SRM, especially for the 4-kHz tonal target conditions. For example, SRM ranged from approximately +6.5 to −6 dB for different listeners when their masked thresholds were compared for the CO−TPS conditions. In general, the difference in listeners’ performances was greatest for the 4-kHz target tone, with the change in thresholds positive for some listeners and negative for others. The between-subjects variability was somewhat smaller (but still high) for the conditions where 500-Hz target stimuli were presented. Paired *t*-tests indicated no significant difference between any two of these three SRM calculations, even without Bonferroni correction of the significance criterion for multiple comparisons with the same (CO) data.

Likewise, the difference between statically panning the target and dynamically panning the target (TPS vs. TPM) essentially resulted in no change in threshold. There was, however, a difference in the masked threshold when ‘diffuse’ maskers were used (TPM${}_{D}-$TS${}_{D}$), where moving the target resulted in a masked threshold that was roughly 2 dB higher than when the target was stationary for 500-Hz targets and about 0.75 dB lower when the target was 4 kHz. Looking at the spread of the individual data for these comparisons reveals that, yet again, variability between listeners was so high as to render these small changes in mean threshold relatively unreliable.

Comparing the TPS−TPM vs. TPM${}_{D}-$TS${}_{D}$ differences in Figure 3 shows a small change in SRM of ≈1 dB, while individuals show values ranging from ≈6.5 to −8.5 dB when the target tone was 4 kHz.

## 4. Experiment 2

Experiment 1 used two maskers placed symmetrically about the listener at $\pm 45^{\circ }$. In so doing, cues such as head shadowing or ‘better-ear effects,’ that have been shown to improve listener performance in spatial release from masking experiments (e.g., see [25,26]) were avoided. Experiment 2 sought to make detection as ‘easy as possible’ so that, if there is some change in auditory detection that results from sound source movement, the results of Experiment 2 might capture that effect.

To this end, a ‘masker fringe’ was added both before and after the stimulus, as this has been shown to improve detection performance as well [20]. Experiment 2 presented pink noise stimuli with temporal characteristics similar to those presented in Braasch and Hartung [27]. Similar to Yost [20], they found that localization of a noise target temporally embedded in a noise masker improved when the masker was presented first and continued on after the target presentation was finished. Braasch [28] speculated that the auditory system capitalized on an ‘old-plus-new’ strategy and showed that modeling based on such a strategy, where the target was subtracted from the masker, could predict their results. If target detection was improved by the difference in location of target and masker, this ‘masker fringe’ stimulus paradigm should improve the chances of observing such an effect.

Localization performance has been found to be poorer when stimulus bandwidth is very narrow [29]. Therefore, the tones presented in Experiment 1 may not have presented as strong a localization cue for target motion as is necessary for any change in detection to occur. If the spatial dimension is important to auditory detection, then having multiple auditory bands across which ITDs are similar (e.g., ‘straightness’ [30]) might improve any ability of the auditory system to profit by target (or masker) movements in a detection task, relative to tonal stimuli. Therefore, 1-octave wide and broadband target noises were presented in Experiment 2.

### 4.1. Stimuli

**Figure 4:**
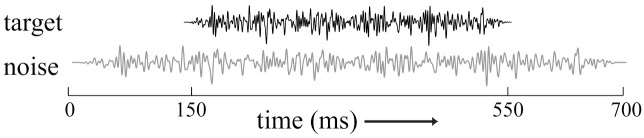
A schematic temporal representation of the stimuli presented in Experiment 2. Details are provided in Section 5.1.

Figure 4 shows a schematic representation of the stimuli for Experiment 2. Stimuli were all filtered pink noises, and all individual stimuli were generated from fresh tokens for every presentation. The masker noise was filtered to 0.3–12 kHz. The target noise was filtered for four different conditions: 0.3–0.6 kHz, 3–6 kHz, 6–12 kHz, and 0.3–12 kHz. The masker noise was 700-ms duration, while the target noise was 400-ms duration, and was temporally centered in relation to the masker so that the first 150-ms of the stimulus was masker-alone, the next 400 ms was target+masker, and the last 150 ms was masker-alone again.

### 4.2. Conditions

Conditions were largely similar to those presented in Experiment 1, but there was only one masker, located at $0^{\circ }$ directly in front of the listener. The location of the target stimulus was varied as follows:

- **CO, Co-located condition**: Target stimulus was co-located with the masker at 0${}^{\circ }$.
- **TPS, Target Panned Static**: Using loudspeakers at at $\pm 30^{\circ }$, the target stimulus was statically panned so that a stationary phantom image would be perceived at either $+20^{\circ }$ or $-20^{\circ }$ (roughly).
- **TPM, Target Panned Moving**: Using loudspeakers at at $\pm 30^{\circ }$, the target stimulus was dynamically panned so that a moving phantom image would be appear to move from approximately $+20^{\circ }$ to $-20^{\circ }$ or vice versa.

### 4.3. Results

**Figure 5:**
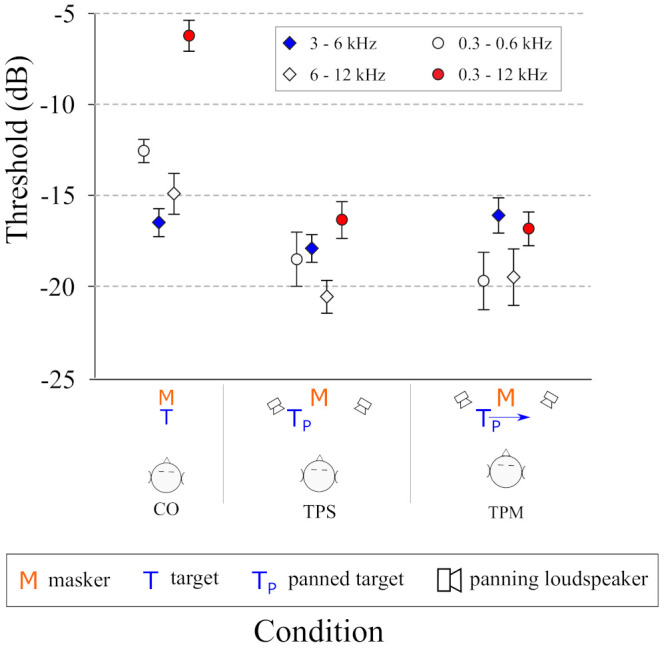
Mean detection thresholds across 10 listeners. Error bars indicate ±1 standard error of the mean. Thresholds were measured the same as in Experiment 1. All conditions are labeled and schematically illustrated along the horizontal axis.

Figure 5 shows the mean detection thresholds averaged across 10 listeners—error bars indicate the standard error of the mean, a measure of between-subjects variability. As in Experiment 1, variability between listeners was very high (since four different target stimuli were presented for each condition, individual data are not shown to retain the legibility of the figure). Planned pairwise *t*-tests (α=0.025, with Bonferonni correction for familywise error) between the the CO condition and the TPS and TPM conditions indicated that there was a statistically significant spatial release from masking for all stimulus frequencies (p<<0.01) except for with the 3–6 kHz target stimulus, where there was essentially no spatial release from masking.

**Figure 6:**
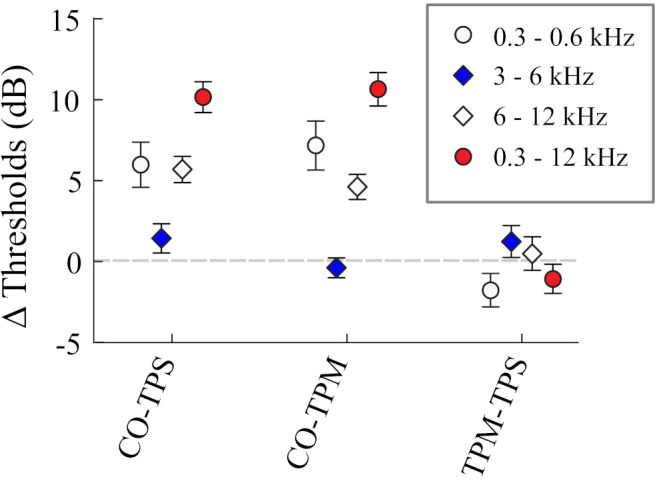
Mean spatial release from masking (SRM), CO−TPS and CO−TPM, and the difference in SRM between the TPS and TPM conditions (TPM−TPS) for 10 listeners. Error bars show the variability between listeners, in terms of the standard error of the mean.

Figure 6 shows the mean calculated spatial release from masking (SRM) across 10 listeners. Planned pairwise *t*-tests between the TPS and TPM conditions, for all stimulus frequencies, were not significant, even without correcting for familywise error. In other words, target movement made no reliable difference in target detection. While the frequency range of the target stimulus affected the absolute thresholds and the release from masking (i.e., CO−TPS and CO−TPM), there was clearly little difference in detection between moving and stationary targets (i.e., TPM−TPS).

## 5. Discussion and Conclusions

### 5.1. Summary

In Experiment 1 we sought to measure spatial release from masking for tonal target stimuli masked by pink noise maskers under conditions where the target and maskers were stationary and when target or maskers were dynamically panned across the listeners’ frontal hemifield to simulate movement of a sound source. A summary of findings is that:

1. The mean spatial release from masking (SRM) across 11 listeners for a 500-Hz tonal target varied from ≈1.75 to 3 dB, depending on how the target location was separated from those of the maskers.
2. There was no appreciable (nor statistically significant) difference in SRM between conditions where the target was stationary vs. dynamically panned to simulate target motion.
3. Using a diffuse noise field as opposed to individual maskers revealed a small difference in detection for moving vs. stationary targets, but the direction of this difference was opposite for target stimuli at 500 Hz vs. 4000 Hz.

Experiment 2 temporally centered the target stimulus in a longer masker to allow listeners to use the change in the masker sound that came when the target was introduced as an additional cue for detection. Only a single masker was presented, from the center loudspeaker at 0${}^{\circ }$. Furthermore, target stimuli were presented at various center frequencies and bandwidths to improve listeners’ ability to detect changes in the spatial cues inherent to the target stimulus.

1. Overall, there was greater SRM for the noise targets than the tonal targets.
2. There was SRM of approximately 5 dB for octave band noise targets (300–600 Hz and 6–12 kHz) and approximately 10 dB for the broadband (0.3–12 kHz) noise targets.
3. Interestingly, there was essentially no SRM for the 3–6 kHz octave-band target noise. We do not currently have an explanation for this, but this lack of SRM was the same regardless of whether the target moved or was stationary.
4. The difference in SRM between stationary and moving targets was not significant, though the mean difference for the 300–600 Hz target noise was approximately 2 dB less masking for the moving target than the stationary target.

### 5.2. Soundfield Spatial Release from Masking in the Literature

Spatial release from masking (SRM) for the tonal stimuli has been extensively studied over headphones (the binaural masking level difference, BMLD). As early as 1948 Licklider [31] and Hirsh [32] noted that detection thresholds for a tonal stimulus masked by a noise over headphones were higher when the target and masker were presented diotically (i.e., $N_{0}S_{0}$) than when the interaural phase of the target was flipped so that it was 180${}^{\circ }$ out of phase at the listener’s two ears (i.e., $N_{0}S_{\pi }$). See [33,34,35]. In these studies the masking level was generally 15–18 dB lower for the out-of-phase target presentation ($N_{0}S_{\pi }$) than it was for the diotic target presentation ($N_{0}S_{0}$).

Surprisingly few studies of spatial release from masking in a soundfield for stimuli presented over loudspeakers have been published. Saberi [36] tested SRM using click trains (as opposed to tones) and found a release from masking of 5–12 dB depending on the relative locations of masker and target (see also [37] for a summary of these and other relevant data). Gatehouse tested for spatial release from masking using a white-noise masker presented from a loudspeaker at $0^{\circ }$ in front of the listener and tones located at 11 different locations at $5^{\circ }$ intervals around the listener in the frontal hemifield. For target tones of 500 Hz, Gatehouse reported an average free-field masking level difference between stimuli presented in quiet vs. when masked by noise maskers of 24 dB. Unfortunately, only a conference abstract survives, so the spatial release from masking in these experiments is unknown. Finally, there is one paper that tested conditions that come closest to those tested in this paper, by Santon [38]. Santon tested for spatial release from masking using 500-ms duration tonal targets and noise maskers. A target tone was presented from $0^{\circ }$ in all conditions. The detection threshold was then measured in quiet, and then with a noise masker presented at 40 dB SPL from a loudspeaker at either $0^{\circ }$, $45^{\circ }$, or $90^{\circ }$. Relative to the masked threshold when both tone and noise were presented from $0^{\circ }$, the masked detection threshold was roughly 5 dB lower when the target was presented from $0^{\circ }$ and the noise masker was presented from $45^{\circ }$. Overall, there have been so few studies for SRM with tones in a sound field (see also [39]) that a thorough determination of the expected size of SRM for the conditions tested in this paper would require additional data.

### 5.3. The Apparent Lack of an Effect on Sound Source Detection from Sound Source Movement

Altogether, we were unable to find any systematic improvement in detection performance across a wide range of stimulus conditions, consistent with the previous findings of Wilcott and Gales [13] and Grantham and Luethke [14] over headphones for low frequency tones and Xiao and Grantham [18] over loudspeakers. Unlike Muñoz et al. [2] for spatial release from masking for speech, we found no real effect on detection for moving maskers over moving targets with tonal target stimuli. In Experiment 2, even fostering a ‘better ear’ advantage by using only one masker and including masker fringes did not elicit any improvement in detection with sound source movement. It seems worth noting that detection of sound source movement may or may not have any effect on detection of a moving sound source. i.e., it may be that sound source movement could have affected detection even if the actual movement of the sound source was not detected.

Target stimulus duration was 400 and 500 ms in Experiments 1 and 2, respectively. The classical tone-in-noise literature generally considers target stimulus duration of ≥100 ms to be ‘long-duration’ (e.g., see [35]), though detection experiments have often been run with far shorter durations (e.g., 25 and 100 ms [40]). Stimuli in the present experiments can therefore be considered ‘long-duration’ target stimuli in terms of sound-source detection.

The relatively slow rate at which the binaural auditory system responds to changes in interaural parameters is often called ‘binaural sluggishness.’ Specifically, listeners have been shown to have difficulty discriminating modulated from unmodulated interaural parameters (usually the ITD) when the rate of fluctuation is greater than approximately 10–50 Hz [41,42], corresponding to a duration for detection of ≈100−20 ms. Similarly, Saberi and Hafter [43] suggested a lowpass filtering model of detection of changing interaural information that occurs during sound source movement with a time constant of approximately 60–130 ms. The stimulus durations used in the current experiment were chosen to be unlikely to be affected by issues of binaural sluggishness.

Grantham [11] found that the minimal audible movement angle (MAMA) was approximately 1 degree across stimulus levels for broadband noise presented by a moving loudspeaker in an anechoic room. For 500-Hz tones the MAMA was around 4 degrees for stimulus durations of approximately 150 ms and higher. Little change in the MAMA was observed for longer stimulus durations.

Taken together, the literature on perceived sound source movement suggests that the duration of the target stimuli presented in Experiments 1 and 2 was several fold that required for listeners to detect auditory motion (as was also determined in the pilot testing for Experiment 1). As such, we believe the stimulus was more than long enough to elicit an effect of sound source motion on sound source detection, but future experiments could of course test this with longer stimuli.

The speed at which the perceived sound source moved may also have had some effect on listener detection of the sound source. The speed of movement in Experiments 1 and 2 was $80^{\circ }$/s. Given the 5-foot radius of the loudspeaker array, this corresponds to a sound source movement of 4.76 miles/hour (7.66 kilometers/hour). Given that people generally walk at approximately 2–4 mph, this speed does not seem very high. Nevertheless, future studies might try faster and slower sound source movements, as only one rate of sound source movement was tested in Experiments 1 and 2. See [44] for more on the perception of sound-source motion and [12] for an excellent, in-depth review of perceived sound-source motion under headphone, loudspeaker, and virtual conditions.

### 5.4. Limitations and Future Experiments

Only a few conditions were tested in the present experiments. Many other configurations of target and maskers, their durations and their relative movements, could be imagined that could then be tested in future work. We were surprised to find that SRM with 3–6 kHz noise targets was not observed while noise targets with lower and higher frequency bands had 5–10 dB SRM. Further research, and some motivating hypothesis for such research, seems in order. Also, while the rate of sound-source motion tested in these experiments seemed reasonably representative of the motion of, for example, a walking talker, Carlile and Best [44] tested for the discrimination of sound-source velocity at lower rates of 15${}^{\circ }$, 30${}^{\circ }$, and 60${}^{\circ }$ per second—future studies could test for an effect on sound-source detection using these slower rates of sound-source motion. Likewise, future studies could try longer stimulus durations, in case more time is required for an effect of sound source movement to emerge. Therefore, the findings presented in this paper are tentative and may not generalize to all sound-source movement conditions.

## Data Availability

Please email Torben.Pastore@asu.edu for data availability.
